# Identification of different lineages of measles virus strains circulating in Uttar Pradesh, North India

**DOI:** 10.1186/1743-422X-9-237

**Published:** 2012-10-16

**Authors:** Akhalesh Kumar Shakya, Vibha Shukla, Harjeet Singh Maan, Tapan N Dhole

**Affiliations:** 1 Department of Microbiology, Sanjay Gandhi Postgraduate Institute of Medical Sciences, Raebareli Road, Lucknow, 226 014, India

**Keywords:** India, Epidemiology, Outbreak, Measles virus, Genotype D8

## Abstract

**Background:**

Genetic analysis of measles viruses associated with recent cases and outbreaks has proven to bridge information gaps in routine outbreak investigations and has made a substantial contribution to measles control efforts by helping to identify the transmission pathways of the virus.

**Materials and methods:**

The present study describes the genetic characterization of wild type measles viruses from Uttar Pradesh, India isolated between January 2008 and January 2011. In the study, 526 suspected measles cases from 15 outbreaks were investigated. Blood samples were collected from suspected measles outbreaks and tested for the presence of measles specific IgM; throat swab and urine samples were collected for virus isolation and RT-PCR. Genotyping of circulating measles viruses in Uttar Pradesh was performed by sequencing a 450-bp region encompassing the nucleoprotein hypervariable region and phylogenetic analysis.

**Results and conclusion:**

Based on serological results, all the outbreaks were confirmed as measles. Thirty eight strains were obtained. Genetic analysis of circulating measles strains (n = 38) in Uttar Pradesh from 235 cases of laboratory-confirmed cases from 526 suspected measles cases between 2008 and 2011 showed that all viruses responsible for outbreaks were within clade D and all were genotype D8.

Analysis of this region showed that it is highly divergent (up to 3.4% divergence in the nucleotide sequence and 4.1% divergence in the amino acid sequence between most distant strains). Considerable genetic heterogeneity was observed in the MV genotype D8 viruses in North India and underscores the need for continued surveillance and in particular increases in vaccination levels to decrease morbidity and mortality attributable to measles.

## Introduction

Measles is a highly contagious and acute febrile illness caused by measles virus, a member of the genus *Morbillivirus*, family *Paramyxoviridae*. It is a childhood disease that causes great morbidity and mortality throughout the world. Global measles mortality was estimated to have decreased 74%, from 535 300 deaths (95% CI 347 200–976 400) in 2000 to 139 300 (71 200–447 800) in 2010 [1]. Measles continues to be a leading cause of childhood morbidity and mortality in developing countries and an outbreak threat in the majority of countries, despite the availability of an effective vaccine for over 50 years [2]. Failure to deliver at least one dose of the vaccine has been reported as the primary cause of mortality and morbidity in developing countries [2]. Risk for illness and death from measles still exists in countries with variable routine vaccination coverage, as in India, where measles is a significant public health problem.

Despite the fact that measles mortality was reduced by more than three-quarters in all World Health Organization (WHO) regions and some progress has been made by WHO Southeast Asia region, India accounted for 47% of estimated global measles mortality in 2010 due to low vaccination coverage [1].

A systematic review of published Indian literature depicts the median case fatality ratio (CFR) of measles to be 1.6% [3]. In India total number of states are 28, out of which at present only 18 states of India comprising of a meager 38% of the population has immunization coverage of more than 80% for the first dose of measles [4].

Studies from the rural, Semi urban, slum and community revealed poor vaccine coverage along with low vaccine efficacy [5] leading to susceptible populations in which outbreaks occur. A study by Sudfeld and Halsey showed higher CFRs in rural areas compared to urban communities in India [6]. It is estimated that 174000 measles deaths occurred in the Southeast Asian region during 2005, with a substantial proportion of this burden in India [7]. WHO estimates that approximately two-thirds of the global burden of measles deaths, namely 136 000 (range 98000 to 180000), occurred in the SEA Region in 2007, with most of them occurring in India.

A critical component of laboratory surveillance for measles is the genetic characterization of circulating wild-type viruses. Though measles virus is a monotypic virus, the WHO currently recognizes 23 genotypes [8] with 8 clades and one provisional genotype termed d11 [9] with some of these genotypes geographically restricted [10]. In measles endemic countries, one or more endemic genotypes are in continuous circulation, while multiple genotypes are detected among the few cases in countries that have eliminated measles [10,11]. There is lower genetic diversity in areas with low vaccination and the higher diversity seen in a country with higher vaccination is likely due to a sampling bias where more surveillance is undertaken compared to the rest of us.

The United States of America declared the elimination of measles based, in part, on molecular epidemiological data [11-13]. Therefore, establishment of baseline molecular data and continuous monitoring of circulating viral genotypes is an important component of laboratory surveillance for measles. The sequence information obtained from virological surveillance studies has proven to be extremely useful for tracking global transmission patterns and for documenting the interruption of transmission in some countries.

There are few reports on circulating wild type measles genotypes in India. In 2002, circulation of measles genotype D4, D8, and A was reported from Pune, Maharashtra [14]. The circulation of genotypes D4, D7, and D8 in some locations in Tamil Nadu has been reported [15,16]. There were no reports available on molecular characterization of measles virus from Uttar Pradesh, a state of North India. Therefore, this study was conducted in Uttar Pradesh to establish a baseline molecular data about the circulating measles virus genotypes. This study describes the molecular characterization of measles strains isolated during 2009–2011 from rural areas of Uttar Pradesh, India. Since the genetic diversity of measles viruses circulating in a state could be correlated with its immunization coverage, which varies between the states in India, it is essential to establish statewide molecular data of measles viruses.

## Materials and methods

### Epidemiologic data

Descriptive information of measles cases and deaths in this report were obtained from 15 outbreaks of 12 different districts (Balrampur, Shravasti, Bahraich, Bareily, Aligarh, Badaun, Etah, Sitapur, Lucknow, Barabanki, Gonda and Sultanpur). The vaccination history and other relevant information regarding outbreaks were recorded by interviewing the mother or next available member of the family on predesigned questionnaire according to WHO format in local language. In the present study, 526 {330 (62.7%) male and 196 (37.3%) female cases} cases of suspected measles were recruited. The total population of children at risk in the outbreak areas was 3057, aged 0–23 years. Recruitment bias was minimized by visiting house to house survey. The cases were recruited into the study after obtaining institutional (SGPGIMS, Lucknow) ethical clearance and written informed consent from the guardians. A copy of the written consent is available for review by the Editor-in-Chief of this journal.

Population denominators for calculation of incidence and mortality rates were determined on the basis of data reported by the SMO (Surveillance Medical Officer) of district health care centers, were outbreaks have reported. Epidemiologic data were analyzed by using Microsoft Excel.

### Specimen collection and virus isolation

A total of 235 blood, 137 urine and 12 throat swabs were collected from 15 outbreaks of 12 different districts mentioned above. The blood was collected between day 4 and 15 of rash onset. The urine and throat swab were collected between 0 and 22 days of rash onset. The blood samples were collected from clinically suspected measles outbreaks (based on the WHO case definition) for the detection of measles specific IgM positive cases. Serological confirmation of measles virus infection in outbreak cases was carried out by immunoglobulin M(IgM) enzyme- linked immunosorbent assay (ELISA) with a WHO recommended kit (Dade Behring) in accordance with the manufacturer’s instructions. Briefly, Reagents were equilibrated at room temperature before the assay was started. Sera, plasma samples or anti-MV references (positive and negative controls) were pre-diluted 1:21 in sample buffer (20 μl + 400 μl). Rheumatoid factor (RF) absorbent was reconstituted in 5 ml H_2_O and 200 μl of the diluted sample were added to an equal volume of RF absorbent (1:42) to precipitate IgG and IgG-linked IgM-RF, which could interfere with IgM detection. Pre-diluted anti-MV reference sera were not treated with RF absorbent. After an incubation time of 15 min at room temperature (18° - 25°C), 150 μl of each sample were placed into a well coated with MV-antigen and one well coated with control-antigen. The reference control P/N (negative control) was added to one pair of wells at the start of the series (wells A1/A2), whereas the reference control P/P (positive control) was loaded into the second (wells B1/B2) and last pair of wells of each plate. For the transfer of the pre-diluted sera and plasma samples an 8-channel pipette was used and samples were mixed thoroughly after dispensing. Plates were covered with an adhesive foil and incubated for 60 min at 37°C. Samples were then washed with no delay. Washing solution was not introduced too quickly into the wells since excessive foam formations had to be avoided. Washing solution was thoroughly aspirated. 180 μl washing buffer was added to each well and aspirated after allowing the buffer to react for 1 – 2 min. Washing was repeated 4 times. Then, 100 μl conjugate buffer (250 μl of anti-human IgM/POD conjugate in 12.5 ml conjugate buffer) was added to each well. The test plate was again covered with an adhesive foil and incubated for another 60 min at 37°C. Washing was repeated as described above. Thereafter, substrate buffer (1 ml chromogen in 10 ml substrate buffer) was distributed to each well. The test plate was incubated for further 30 min at room temperature and after adding 100 μl stop solution to each well, absorbance was measured in the photometer at the reference wavelength of 450 nm. For each test and reference sample the difference in absorbance (∆A) in O.D. of the value obtained for MV-antigen and control-antigen (∆A = A_antigen_ – A _control__antigen_) was calculated. Results were only used for further evaluation if the following validation criteria were met: The ∆A-values for each pair of reference P/P wells must be within the range defined by the lower and upper margins given by the manufacturer. In addition, the ∆A-values of the individual readings of the reference P/P at the start and the end of the series must not deviate from the mean of these two readings by more than ± 20% and each pair of reference P/P wells must reach or exceed a value of 0.2 O.D. The ∆A for the reference P/N should always be less than 0.1 O.D. The reference P/P is not only used for validation of the test, but is also necessary for the calculation of a correction factor. The nominal value given in the enclosed table of values for the reference P/P was divided by the mean value of its ∆A values obtained in the test. The ∆A-values of the samples, determined in the same series, were corrected by multiplying with this factor and the cut-offs for negativity (> 0.1 O.D.) and positivity (< 0.2 O.D.) were applied as recommended by the manufacturer.

The urine samples and throat swabs were directly inoculated on Vero/hSlam cell line for isolation of measles virus. Vero/hSlam cells were obtained from National Institute of Virology, Pune, India. The Vero cells are transfected with a plasmid encoding gene for the human SLAM (signaling lymphocyte-activation molecule, also designated CDw150) molecule [17].

Each specimen was individually inoculated at a volume of 0.5 ml into a 25 cm^2^ plastic tissue culture flask containing a sub-confluent monolayer of Vero/hSLAM cells. Each sample was inoculated in duplicate and uninoculated flasks were used as negative controls. The flasks were incubated in a 37°C humidified incubator and examined daily for cytopathic effects (CPE). CPE was observed as giant multinucleated syncytium formation and detachment of cells. Blind passages were done for those not showing CPE after 7 days. The presence of Measles virus (MV) was confirmed by reverse transcriptase polymerase chain reaction (RT-PCR). At the level of 75-100% CPE, the cultures were harvested and stored at -70°C for further use.

### RNA Extraction and RT-PCR

RNA was extracted from 250 μl of infected cell lysate or clinical samples using a Trizol reagent, following the manufacturer's instructions. RT-PCR amplification was performed using previously described primers to amplify a 600 bp fragment in the N gene which included the 450 bp fragment recommended for genotyping [12]. PCR products were purified using the QIA quick gel extraction kit (QIAGEN). Sequences of the PCR products were derived by automated sequencing and the Big Dye terminator v3.0 chemistry according to the manufacturer's protocol in both sense and antisense strands by an automated ABI PRISM™ 3100 DNA Sequencer (Perkin Elmer). Sequence proof reading and editing was conducted with Sequencer™ (Gene Codes Corporation). Sequence data were analyzed using version 7.0 of Bioedit and phylogenetic analyses were performed using Bioedit (http://www.mbio.ncsu.edu/BioEdit/bioedit.html) and Mega5.1 (http://www.megasoftware.net). The robustness of the groupings was assessed using bootstrap resampling of 1000 replicates and the trees were visualized with Mega programs. Nucleotide sequence data from 38 strains were deposited in GenBank under accession numbers: JN381167-JN381188, HQ453170- HQ453179, HQ141406- HQ141408, HM146188, HM146189, and GU561991.

## Results

### Epidemiological findings

**Figure 1:**
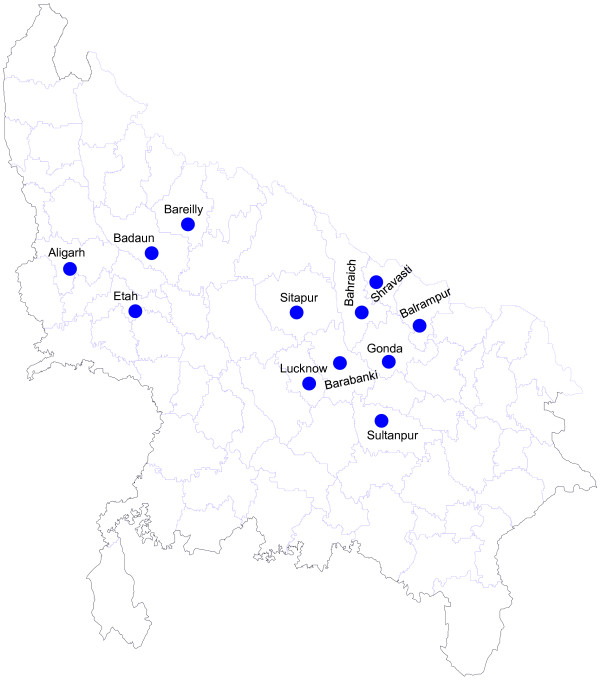
Geographical distribution of measles genotype D8 obtained during outbreaks.

A total number of 15 outbreaks from 12 different districts were investigated during the study period between 2008 and 2011 (Figure 1). Standard WHO measles case definition was followed to clinically diagnose the measles cases. Maximum numbers of cases were reported from District Balrampur (60 cases), Shravasti (98 cases), and Bahraich (45 cases).

A total of 526 clinically confirmed cases were reported during the study period with a mean age of 6.791 ± 4.725 years, ranging from 6 months to 23 years. The median age was 6 years and the male to female ratio were 1.68:1. The highest incidence has been reported in the 180 cases aged 1–5 years (34.2%) of age group. The immunization history shows that out of 526 measles infected cases only 26 (4.9%) cases were found to be vaccinated while the remainders were unvaccinated or were not able to recall vaccination history. More males were vaccinated compared to females.

Totally, 235 sera were tested for the presence of measles specific IgM as per manufacturer’s recommendation. 227 sera sample (97%) were positive for measles IgM and 8 sera samples (3%) were equivocal. A total of 142 male cases and 93 female cases were found positive. From the clinically suspected cases, 188 male and 103 female cases were not tested for IgM due to refusal of sample collection from their parents/guardians.

MV isolation from urine sample and throat swab was attempted in Vero/hSLAM cell line. Only 15% (20) samples were positive by cell culture.

RT-PCR analysis shows only 28.46% (38 samples) positivity. All the 38 samples were sequenced.

### Genotyping results

**Figure 2:**
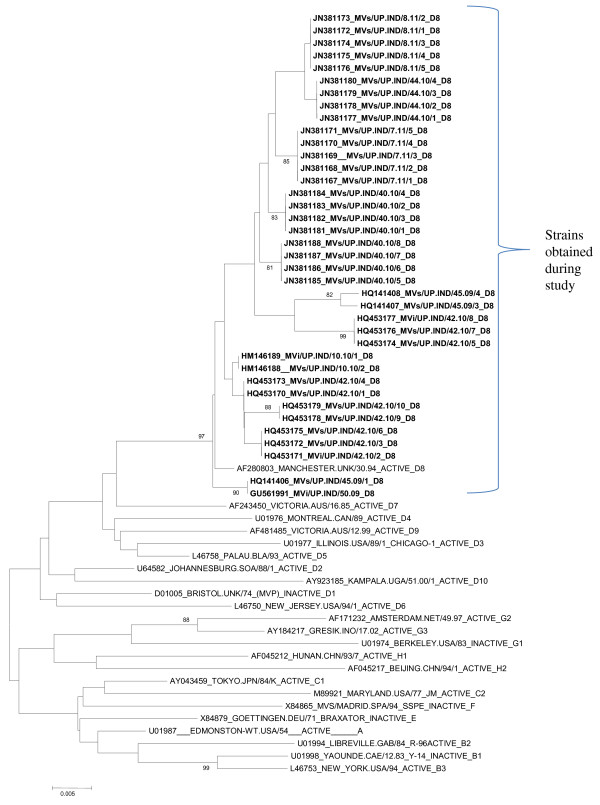
**Phylogenetic analysis of measles virus genotypes D8 strains with the WHO reference strains.** Scale bar indicates base substitutions per site.

Phylogenetic analysis of measles strains obtained during the study with WHO reference sequences revealed that all the Uttar Pradesh strains segregated with clade D, genotype D8 measles strains. The sequence identity of D8 genotypes with the WHO reference genotype D8 was ranged from 97.1% to 99.6%. The phylogenetic analysis of all the 38 D8 sequences among themselves reveals that these sequences show different lineages (Figure 2). According to our findings, these different lineages showed that the virus responsible for the different outbreaks were different. The identity of two strains (HM146188, HM146189) with the reference genotype D8 was 99.6%. Two strains (HQ453170, HQ453173) were 99.3% identical with reference strain. Ten strains (JN381167, JN381168, JN381169, JN381170, JN381171, HQ453171, HQ453172, HQ453175, HQ141406, and GU561991) were 99.1% identical to the reference strain. Six strains (JN381185, JN381186, JN381187, JN381188, HQ453178, and HQ453179) were 98.7% identical with the reference strain. Five strains (JN381172, JN381173, JN381174, JN381175, and JN381176) were 98.5% identical with the reference strain. One strain (HQ141407) was 98.2% identical. Eight strains (JN381177, JN381178, JN381179, JN381180, JN381181, JN381182, JN381183, and JN381184) were 98.0% identical with the reference strain. One strain (HQ141408) was 97.6% identical with the reference strain. Remaining three strains (HQ453174, HQ453176, and HQ453177) were 97.1% identical with the reference strain.

Out of the 38 D8 strains, eleven strains (JN381167, JN381168, JN381169, JN381170, JN381171, HQ453170, HQ453173, HM146188, HM146189, HQ141406, and GU561991) exhibited 98.7% amino acid sequence identity with WHO reference strain. Another eleven strains (JN381172, JN381173, JN381174, JN381175, JN381176, HQ453174, HQ453176, HQ453177, HQ453178, and HQ453179, HQ141407) showed 97.3% amino acid sequence identity with the reference strain. Seven strains (JN381185, JN381186, JN381187, JN381188, HQ453175, HQ453171 and HQ453172) showed 98% amino acid sequence identity. Eight strains (JN381177, JN381178, JN381179, JN381180, JN381181, JN381182, JN381183 and JN381184) showed 96.7% amino acid identity with the reference strain. Only one strain (HQ141408) revealed 96% amino acid identity with the reference strain.

### Intra Genotypic variations (Genotype D8)

The genotype D8 detected in the present study was differed from each other by maximum of 3.4% while some strains were 0.2% differ from each other. The Genotype D8 strains obtained from present study were differed from WHO reference strain at 20 different positions. Of these only 11 substitutions leads to amino acid changes and remaining 9 substitutions were silent mutation. All the 38 strains have nucleotide substitution at one position at 1564 (A → G) while maximum number of strains have nucleotide substition at nine positions at 1152(C → T), 1188(G → A), 1225 (G → T), 1269(T → C), 1418(T → C), 1154(C → T), 1183(C → T), 1490(G → C), and 1525(G → A). All the 38 strains exhibited substitution at 1564 nucleotide position (A → G) which leads to amino acid changes from N(Asparagine) to D (Aspartic acid). Nucleotide substitution at position 1225 (G → T) which leads to amino acid change from A (Alanine) to S (Serine). Nucleotide substitution at position 1418 (T → C) leads to amino acid change from L (Leucine) to P (Proline). Nucleotide substitution at position 1154 (C → T) leads to amino acid change from P (Proline) to L (Leucine). Nucleotide substitution at position 1183 (C → T) leads to amino acid change from R (Arginine) to T (Threonine). Nucleotide substitution at position 1525 (G → A) leads to amino acid change from G (Glycine) to S (Serine).

### Genetic relatedness of Uttar Pradesh genotype D8 measles strains to Indian measles strains

**Figure 3:**
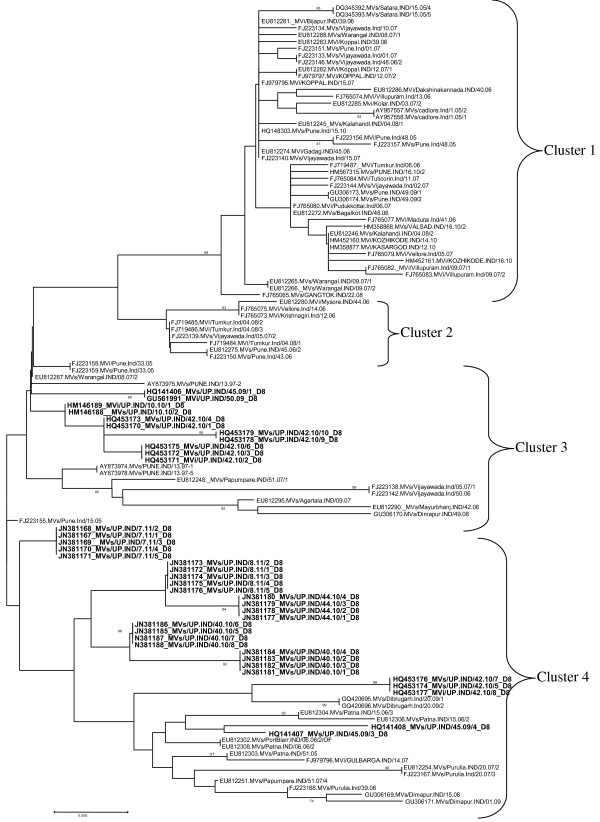
**Phylogenetic analysis of measles virus genotypes D8 strains obtained with the other parts of India.** Scale bar indicates base substitutions per site. Strains obtained during study are shown as bold.

In this analysis 38 sequences obtained from present study were compared with the sequences reported from other parts of India. The phylogenetic analysis of all strain revealed that all the strains were grouped into four clusters. Sequences obtained from present study were grouped into clusters 3 and 4 and none of the sequence grouped into cluster 1 and 2. Cluster 3 contains the strains reported from Pune, Papumpare, Mayurbhanj, Dimapur, Agartala Vijaywada and Warangal. Cluster 4 contains the strains reported from Papumpare, Dibrugarh, Dimapur, Purulia, Patana, Gulbarga and Portblair (Figure 3).

In cluster 3 the sequences from present study shows 99.6% to 98.7% similarity with the Pune strain isolated in 2005. The similarity with the Warangal strain was 99.8% to 98.9%. The similarity with the Papumpure strain was 98.7% to 97.8%. Similarity with Agartala strain was 99.3% to 97.1%. Similarity with Dimapure strain was 97.4% to 96.5%. Similarity with the Vijaywada strain was 97.6% to 96.7%.

In cluster 4 the sequences from present study shows 98.7% to 97.8% similarity with the Papumpare strain. The similarity with the Purulia strain was 98.7% to 96.5%. The similarity with the Protblair strain was 98.2% to 97.4%. Similarity with Patna strain was 98.2% to 97.4%. Similarity with Dimapure strain was 97.4% to 96.7%. Similarity with the Dibrugarh strain was 98.7% to 97.6%.

**Figure 4:**
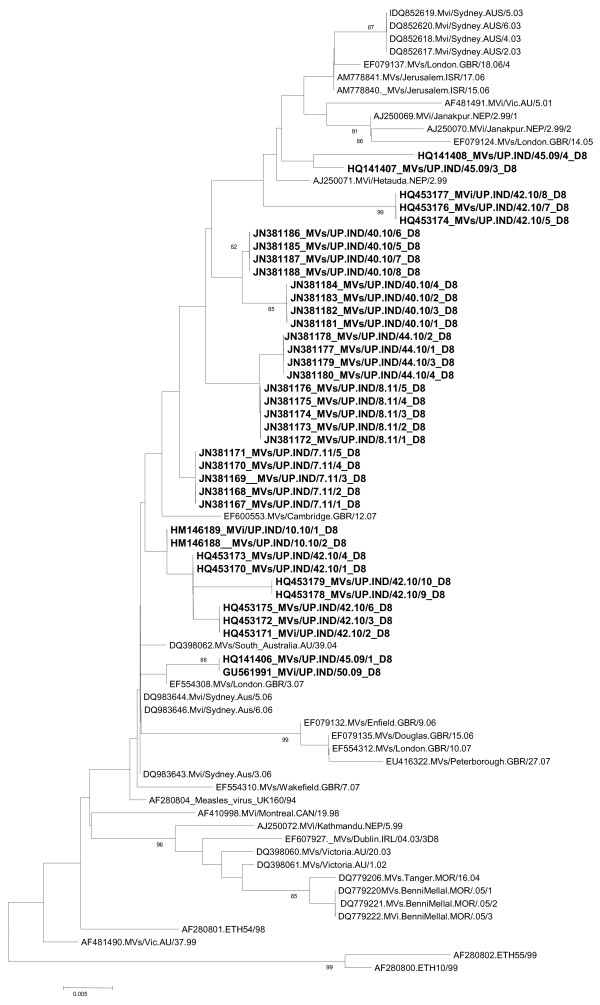
**Phylogenetic analysis of measles virus genotypes D8 with the global D8 genotypes.** Scale bar indicates base substitutions per site. Strains obtained during study are shown as bold.

The strains JN381167_ JN381171 were 0.9% diverse with the Cambridge strain. While the strains JN381177_ JN381184 were 2.0% diverse with the Cambridge strain. The strains JN381172_ JN381176 were 1.5% diverse with Cambridge strain. The strains JN381185_ JN381187 were 1.8% diverse with Cambridge strain (Figure 4).

HM146188 and HM146189 strains have the 99.8% similarity with Sydney strain. These two strains also have the 99.6% similarity with London strain.

## Discussion

There are few reports on circulating wild type measles genotypes in India. In 2002, circulation of measles genotype D4, D8 and A was reported from Pune, Maharashtra [14]. The circulation of genotypes D4, D7 and D8 in some locations in Tamil Nadu has been reported [15,16]. There were no reports available on molecular characterization of measles virus from Uttar Pradesh a state of North India. Therefore, this study was conducted in Uttar Pradesh to establish a baseline molecular data about the circulating measles virus genotypes. Since the genetic diversity of measles viruses circulating in a state could be correlated with its immunization coverage, which varies between the states in India, it is essential to establish statewide molecular data of measles viruses.

In the present study 38 strains were obtained from 15 villages of 12 districts. All the strains were obtained from the villages. The studied population in the present study comprised of 3507 children. The known vaccination status of studied population was only 28%. Among the 526 cases investigated, 26 (4.9%) cases were immunized (as evidenced by availability of immunization card or statement of parents/ health workers). Immunization status was not known for 500 (95%) cases or not able to recall vaccination history. More males were vaccinated compared to females in the present study. In the present study the majority of cases were of lower socioeconomic status, illiterate and less aware of vaccination programs. A significant high severity of symptoms has been found in another study in cases belonging to the lower socioeconomic status [18], especially among illiterates; an indirect indicator of poor hygiene and awareness for the vaccination program.

In our study, we also found that sex, maternal literacy, social category, maternal occupation, and standard of living were important “demand-side” predictors in the immunization status of children, which was found in other studies as well [19-21]. While some studies [22] have shown a significant role of health workers in reducing sex bias, we found that, despite adjusting for the role of health services and presence of health workers, girls are less likely to be immunized than boys.

A higher proportion of males were affected in the present study as compared to the females which is corroborated by few other studies [23,24]. The recruitment bias was minimized due to house to house survey. The possible reason for more males get infected could be the differential attitude of parents towards female child or gender difference in the outbreak area.

All the blood samples were collected between 4 and 15 days of rash onset. The blood samples were collected from clinically suspected measles outbreaks (based on the WHO case definition). From the results of measles IgM positivity based on the timing of sample collection, it was noticed that 227 samples (97%) and 8 samples (3%) that were collected between days 4 and 15 after the onset of rash were positive and equivocal respectively. The timing of sample collection is probably responsible for negativity in epidemics. The optimum time for blood sample collection for IgM detection is 4–28 days post onset of rash [25,26]. This was also confirmed in the present study. We also noticed such finding by other Indian researchers where it is well documented about 100% positivity for IgM ELISA where blood samples were collected between days 4 and 22 after the onset of rash, where as samples collected between days 1 and 3, 17% positivity were noticed [15].

Virus isolation from urine sample attempted in the Vero/hSlam cell line yielded virus from 20 samples (15%). Similar low success rates were observed in previous studies, were 12% [15] and 18% [27] yields were found. RT-PCR analysis shows only 28% (39 samples) positivity.

Reason for low yield of virus isolation from cell culture and RT-PCR could be timing of sample collection and transportation of the samples to the laboratory. Previous finding clearly demonstrated that percentage of virus isolation varied based on timing of sample collection. The urine samples collected between 8–13 days shows 33% positivity and sample collected between 14 to 20 days shows 29% positivity [28]. All the positive samples reported in the present study by cell culture and RT-PCR analysis was collected within 6 days of rash onset. All the samples collected beyond 6 days were negative.

The percentage of virus isolation also varied based on cell culture and RT-PCR analysis. The high percentage positivity by RT-PCR as against virus isolation was also reported previously from India [14,15]. Study from Suburban Khartoum [29] also showed high percentage positivity by RT-PCR compared to virus isolation.

All these findings indicate the fact that the optimum concentration of virus and timing of collection of sample is required for the isolation of the virus and RT-PCR is more sensitive as compared to virus isolation.

Optimally, measles virus is excreted from infected cases only for the first 5–7 days after rash onset, often in low titers. WHO has recommended samples for virus isolation should be collected within 5 days after rash onset [26]. For these reasons, attempts to detect virus from suspected measles cases after a week of rash onset is not considered to be a useful diagnostic tool.

It is well understood that measles case confirmation by virus isolation is less sensitive. The measles virus genome is relatively stable and shows minor detectable changes over the course of an outbreak or even over 12 months. Hence, isolation of virus from all cases is not considered necessary and 1 or 2 isolates from each outbreak or chain of infection will provide sufficient data to determine transmission pathways [30].

During the study period genotype D8 found to be circulating in Uttar Pradesh India. All the cases reported in the present study did not have travel history or contact with traveler, suggesting that these viruses are indigenous.

The genotype D8 detected in the present study was differed from each other by maximum of 3.4% while some strains were 0.2% differ from each other. Genetic heterogeneity of the Indian measles viruses is not a result of increased mutation rates but is due to the presence of multiple co-circulating lineages of viruses within the endemic region.

This analysis clearly demonstrated that multiple lineages of genotype D8 are co-circulating and disseminated widely throughout the state, a pattern consistent with an endemic genotype.

Though measles surveillance in India is in its infancy, during the preceding 15 years only Clade D genotypes (D4, D7, D8) have been detected in India, whereas surrounding countries have detected D4, D7, D8, D9, D5, H1, d11, and G3 genotypes [9,31]. These external genotypes were not imported into India between 1995 and 2010. The possibilities of missing genotypes or of importation of other genotypes must be further studied and documented with continuous countrywide molecular surveillance in India. Other countries have also documented co-circulation of genotypes, probably due to multiple chains of transmission during an outbreak [32,33].

The mutation rate amongst field isolates of measles virus is low and appears to be random rather than driven by vaccine pressure or immune responses. Within a genotype, nucleotide difference (virus lineage) can assist in distinguishing separate episodes of transmission [34]. In the present study the nucleotide difference was observed based on the outbreak / transmission chain. In countries or regions with endemic measles, many lineages of a single genotype may co- exist; however as countries begin to move from endemic to epidemic measles the diversity of sequences within the circulating genotypes decreases [35-38]. This is consistent with the present study also; multiple lineages of genotype D8 strains were circulating in the state during 2008 – 2011. However, in 2002, Oliveira MI et al. have reported, the genotype D6 virus associated with a large measles outbreak that occurred in several South American countries between 1996 and 1997 had identical N gene sequences suggesting rapid spread of a single lineage [39]. Analysis of measles viruses circulating in Burkina Faso, before and after a mass vaccination campaign, showed that the number of circulating lineages was greatly reduced following the campaign. Sequence analysis of viruses isolated from outbreaks that occurred after the vaccination campaign suggested that virus was introduced from a single source [38].

## Competing interests

The authors have no commercial affiliations or conflict of interest to declare.

## Authors’ contributions

AKS and VS carried out all experiments, analysis and drafted the manuscript. HSM helped in the data analysis. TND participated in the design of the study and helped to draft the manuscript. All the authors have read and approved the final manuscript.
